# Supplementary material for: Dynamic acoustic-to-categorical representations of phonemes and prosody along ventral and dorsal speech streams
Source: Nat Commun. 2026 Jul 10;17:6082. doi: 10.1038/s41467-026-75240-0 (PMC13354773; doi:10.1038/s41467-026-75240-0)
Supplement: Supplementary file 2 — Reporting Summary [file 41467_2026_75240_MOESM2_ESM.pdf]

nature portfolio

Corresponding author(s): Dr. Daniela Sammler

Last updated by author(s): Jun 11, 2026

Reporting Summary

Nature Portfolio wishes to improve the reproducibility of the work that we publish. This form provides structure for consistency and transparency in reporting. For further information on Nature Portfolio policies, see our Editorial Policies and the Editorial Policy Checklist.

Statistics

For all statistical analyses, confirm that the following items are present in the figure legend, table legend, main text, or Methods section.

n/a	Confirmed	
☐	☒	The exact sample size (*n*) for each experimental group/condition, given as a discrete number and unit of measurement
☐	☒	A statement on whether measurements were taken from distinct samples or whether the same sample was measured repeatedly
☐	☒	The statistical test(s) used AND whether they are one- or two-sided<br>*Only common tests should be described solely by name; describe more complex techniques in the Methods section.*
☐	☒	A description of all covariates tested
☐	☒	A description of any assumptions or corrections, such as tests of normality and adjustment for multiple comparisons
☐	☒	A full description of the statistical parameters including central tendency (e.g. means) or other basic estimates (e.g. regression coefficient) AND variation (e.g. standard deviation) or associated estimates of uncertainty (e.g. confidence intervals)
☐	☒	For null hypothesis testing, the test statistic (e.g. *F*, *t*, *r*) with confidence intervals, effect sizes, degrees of freedom and *P* value noted<br>*Give P values as exact values whenever suitable.*
☒	☐	For Bayesian analysis, information on the choice of priors and Markov chain Monte Carlo settings
☐	☒	For hierarchical and complex designs, identification of the appropriate level for tests and full reporting of outcomes
☐	☒	Estimates of effect sizes (e.g. Cohen's *d*, Pearson's *r*), indicating how they were calculated

Our web collection on statistics for biologists contains articles on many of the points above.

Software and code

Policy information about availability of computer code

Data collection	MEG (magentoencephalography) data were acquired using a Neuromag Vectorview system (MEGIN) with 306 channels (102 magnetometers and 204 gradiometers) in a magnetically shielded room in Bennewitz, Germany. During the recording, we used the Presentation® software (version 18.0, Neurobehavioral Systems) to present visual and auditory stimuli and to collect individual participants' behavioural responses.
Data analysis	Behavioural data were curated and analysed using MATLAB (R2021a, MathWorks). The statistical testing of the behavioural data at the group level was conducted using statsmodels (version 0.13.2) and pingouin (for computing effect sizes, version 0.5.4) toolboxes based on Python 3.10.5.<br>MEG data were analysed in a Python environment (version 3.10.5). For the preprocessing of the MEG data, we primarily used MNE-Python (version 1.1.0). To remove the line noises and its harmonics, we employed dss_line() function (i.e., ZapLine) in meegkit (version 0.1.2). After segmenting the MEG data into trials, bad epochs were automatically identified and rejected using autoreject (version 0.3.1).<br>For source reconstruction, individual structural MRI (magnetic resonance imaging) data were preprocessed using FreeSurfer (version 7.3.2). The MEG-MRI coregistration and source reconstruction were implemented in MNE-Python (version 1.1.0).<br>The computation of neural RDMs (representational dissimilarity matrices) in the ROIs (regions of interest) and searchlights were performed with custom code adapting mne-rsa (version 0.8dev.) and rsatoolbox (version 0.1.3).<br>For time-resolved RSA (representational similarity analysis), we used custom code adapting mne-rsa (version 0.8dev.) and scikit-learn (version 1.1.2) for implementing non-negative least squares regression.<br>mTE (multivariate transfer entropy) analysis was performed using custom Python code by translating and adapting the original MATLAB code at the following Github repository: https://github.com/ide2704/Kernel_Renyi_Transfer_Entropy/tree/master.<br>The statistical inference on the neural data was conducted using MNE-Python (version 1.1.0).<br>The code supporting the ROI-based time-resolved RSA and mTE analysis, as well as the creation of the main figures (Figs. 1-6) are publicly

available in the following Github repository: https://github.com/SeungCheolBaek/representation_dynamics_phonemes_prosody (https://doi.org/10.5281/zenodo.20509067).

For manuscripts utilizing custom algorithms or software that are central to the research but not yet described in published literature, software must be made available to editors and reviewers. We strongly encourage code deposition in a community repository (e.g. GitHub). See the Nature Portfolio guidelines for submitting code & software for further information.

Data

Policy information about availability of data

All manuscripts must include a data availability statement. This statement should provide the following information, where applicable:

- Accession codes, unique identifiers, or web links for publicly available datasets
- A description of any restrictions on data availability
- For clinical datasets or third party data, please ensure that the statement adheres to our policy

We have provided the stimuli presented and the behavioural responses of individual participants collected during the MEG experiment, as well as the preprocessed neural data in source space, including the neural RDMs and time-resolved RSA and mTE results based on the ROIs in the public Github repository as follows: https://github.com/SeungCheolBaek/representation_dynamics_phonemes_prosody (https://doi.org/10.5281/zenodo.20509067).

Research involving human participants, their data, or biological material

Policy information about studies with human participants or human data. See also policy information about sex, gender (identity/presentation), and sexual orientation and race, ethnicity and racism.

Reporting on sex and gender	We collected the self-reported biological sex of our participants, as reported in our study. No gender information is collected. No sex- or gender-based analysis was performed, as we were interested in common neural processing of phonemes and prosody, regardless of sex or gender differences.
Reporting on race, ethnicity, or other socially relevant groupings	No information about race, ethnicity, or other socially relevant groupings was collected.
Population characteristics	34 native German speakers (17 females, aged from 20 to 35, mean age=26 years, SD=4) participated in this study. All participants self-reported normal hearing with no history of neurological or psychiatric disorders, and were assessed as right-handed by the Edinburgh Handedness Inventory.
Recruitment	Participants were recruited in Leipzig, Germany from the participant database of the Max Planck Institute for Human Cognitive and Brain Sciences.
Ethics oversight	This study was approved by the Ethics Committee of the Medical Faculty, Leipzig University (403/14-ff). Informed consent was obtained from every participant. Participants were given monetary reimbursement for their participation.

Note that full information on the approval of the study protocol must also be provided in the manuscript.

Field-specific reporting

Please select the one below that is the best fit for your research. If you are not sure, read the appropriate sections before making your selection.

☐ Life sciences ☒ Behavioural & social sciences ☐ Ecological, evolutionary & environmental sciences

For a reference copy of the document with all sections, see nature.com/documents/nr-reporting-summary-flat.pdf

Behavioural & social sciences study design

All studies must disclose on these points even when the disclosure is negative.

Study description	The MEG experiment consisted of six runs, each of which contained four blocks. Across the four blocks, participants were alternately asked to identify either the word (“Bar”/“Paar”) or the prosody (“statement”/“question”) of the mono-syllabic words that gradually varied in five morph levels along two orthogonal dimensions: (i) the voice onset time of the word-initial phoneme shifting from /b/ to /p/, and (ii) the pitch contours of the word shifting from statement (falling) to question (rising). The behavioural data were binary responses collected during the MEG experiment, after one-sixth of the stimuli, randomly distributed across blocks.
Research sample	A total of 34 native German speakers (17 females, aged from 20 to 35, mean age=26 years, SD=4) completed the experiment. Although the sample is not fully representative, it was selected to minimize potential age-related degradation in hearing ability and to ensure familiarity with the German-language stimuli. We acknowledge that this participant pool may be subject to self-selection bias, as volunteers for research participation may differ from non-volunteers in unmeasured ways. However, this is unlikely to have substantially influenced the results, as the study focused on relatively automatic and fundamental aspects of neural speech processing.

Sampling strategy	A sample size of approximately 30 participants has shown to be reliable to detect significant effects in similar experimental designs with 80% power at p=0.05 significant level.
Data collection	The MEG data were acquired using a Neuromag Vectorview system (MEGIN) with 306 channels (102 magnetometers and 204 gradiometers) in a magnetically shielded room in Bennewitz, Germany. The behavioural data consisted of binary responses collected using a response box. During data collection, only the participant and the experimenter was present. Due to a within-participant design, the experimenter was not blinded to the experimental conditions.
Timing	Both behavioural and MEG data were collected between December 2015 and January 2017.
Data exclusions	A total of five participants were excluded from the analysis.<br>Four participants, who showed a difference of less than 0.6 between the minimal and maximal proportion of "Paar" or "question" responses across the five phoneme or prosody levels, were excluded, as they either perceived no clear question/Paar or no clear statement/Bar.<br>One additional participant was excluded for having more than 20% of the preprocessed MEG epochs rejected.
Non-participation	No participants dropped out or declined.
Randomization	The order of task and speaker per experimental run was randomised and counterbalanced across participants.<br>The order of the stimulus presentation were pseudorandomised across runs and participants following the principles of a type-1 index-1 sequence to prevent carry-over and position effects.<br>The response button assignment was randomised in each response trial and balanced across stimuli, tasks, and speakers.

Reporting for specific materials, systems and methods

We require information from authors about some types of materials, experimental systems and methods used in many studies. Here, indicate whether each material, system or method listed is relevant to your study. If you are not sure if a list item applies to your research, read the appropriate section before selecting a response.

Materials & experimental systems

n/a	Involved in the study
☒	☐ Antibodies
☒	☐ Eukaryotic cell lines
☒	☐ Palaeontology and archaeology
☒	☐ Animals and other organisms
☒	☐ Clinical data
☒	☐ Dual use research of concern
☒	☐ Plants

Methods

n/a	Involved in the study
☒	☐ ChIP-seq
☒	☐ Flow cytometry
☐	☒ MRI-based neuroimaging

Plants

Seed stocks	*Report on the source of all seed stocks or other plant material used. If applicable, state the seed stock centre and catalogue number. If plant specimens were collected from the field, describe the collection location, date and sampling procedures.*
Novel plant genotypes	*Describe the methods by which all novel plant genotypes were produced. This includes those generated by transgenic approaches, gene editing, chemical/radiation-based mutagenesis and hybridization. For transgenic lines, describe the transformation method, the number of independent lines analyzed and the generation upon which experiments were performed. For gene-edited lines, describe the editor used, the endogenous sequence targeted for editing, the targeting guide RNA sequence (if applicable) and how the editor was applied.*
Authentication	*Describe any authentication procedures for each seed stock used or novel genotype generated. Describe any experiments used to assess the effect of a mutation and, where applicable, how potential secondary effects (e.g. second site T-DNA insertions, mosiacism, off-target gene editing) were examined.*

Magnetic resonance imaging

Experimental design

Design type	The MRI scanner was used to acquire only structural images.
Design specifications	Individual structural scans were collected during a single scanning session that lasted about 10 min. including the preparation.
Behavioral performance measures	No task was involved during the structural MRI scanning.

Acquisition

Imaging type(s)	T1-weighted images
Field strength	3T
Sequence & imaging parameters	Magnetization-prepared rapid gradient-echo (MP-RAGE) pulse sequence was used (TR = 2300 ms, TE = 5.52 ms, 9 degree flip-angle, 176 sagittal slices, slice thickness = 1 mm, field of view= 240 x 256 mm, isotropic voxel-size=1 x 1 x 1 mm3).
Area of acquisition	Whole brain scans were acquired.

Diusion MRI ☐ Used ☒ Not used

Preprocessing

Preprocessing software	Structural MRI data were preprocessed using FreeSurfer (version 7.3.2) to reconstruct participants' native cortical spaces.
Normalization	Source reconstructed data were morphed onto FreeSurfer's fsaverage space using MNE-Python (version 1.1.0).
Normalization template	MNI305 coordinate was used for mapping participants' native spaces onto fsaverage cortical space.
Noise and artifact removal	Incorrect tissue segmentations by FreeSurfer were visually inspected and manually corrected.
Volume censoring	No volume censoring was applied.

Statistical modeling & inference

Model type and settings	MRI data were not used for statistical analysis.
Effect(s) tested	*Define precise effect in terms of the task or stimulus conditions instead of psychological concepts and indicate whether ANOVA or factorial designs were used.*

Specify type of analysis: ☐ Whole brain ☐ ROI-based ☐ Both

Statistic type for inference (See Eklund et al. 2016)	*Specify voxel-wise or cluster-wise and report all relevant parameters for cluster-wise methods.*
Correction	*Describe the type of correction and how it is obtained for multiple comparisons (e.g. FWE, FDR, permutation or Monte Carlo).*

Models & analysis

n/a	Involved in the study
☒	☐ Functional and/or effective connectivity
☒	☐ Graph analysis
☒	☐ Multivariate modeling or predictive analysis
