# Supplementary material for: Dynamic acoustic-to-categorical representations of phonemes and prosody along ventral and dorsal speech streams
Source: Nat Commun. 2026 Jul 10;17:6082. doi: 10.1038/s41467-026-75240-0 (PMC13354773; doi:10.1038/s41467-026-75240-0)
Supplement: Supplementary file 3 — Transparent Peer Review file [file 41467_2026_75240_MOESM3_ESM.pdf]

nature portfolio

Peer Review File

Dynamic acoustic-to-categorical representations of phonemes and prosody along ventral and dorsal speech streams

Corresponding Author: Dr Daniela Sammler

This file contains all reviewer reports in order by version, followed by all author rebuttals in order by version.

Version 0:

Reviewer comments:

Reviewer #1

(Remarks to the Author)
Baek and colleagues present a combined MEG and behavioural study of speech perception in which they use time-resolved MEG data to interrogate the neural representations of phonemes and prosodic contours, based on voice-onset-time (/b/ vs. /p/) and pitch contour (question vs. statement), respectively. The analyses delineate findings indicating largely overlapping representations of stimulus acoustics and perceived categories for both phonemes and prosody, as well as some relative distinctions between the two aspects of the spoken words (e.g. representation of prosody but not phonemes in inferior frontal and premotor cortices). A multivariate information transfer analysis revealed further differences in the patterning of notionally bottom-up and top-down transfers between regions of interest for phonemic and prosodic cues, respectively.

I find the paper to be very clearly written and all of the methods and findings to be carefully and clearly expressed. The study design and analysis implementation are rigorous and harness methods effectively to offer new insights onto the dual streams posited to support speech perception in the brain. I only have a couple of comments:

I felt that having the outline of the findings in the opening sections, followed by the detailed reporting of the results, and another summary in the Discussion, made the text feel somewhat dense and repetitive. If possible, a more integrated reporting of the results would help to make the paper more concise and readable.

A more emphatic account of the novelty of this study and greater depth of analysis of the theoretical implications would help readers. For example, the finding that representations of phonemic categories do not extend to premotor and inferior frontal cortex – while prosodic categories do - calls to mind the discussions of around 10-15 years ago around the engagement of motor responses for speech perception, where some studies implicated (pre)motor reponses in the phonemic analysis of spoken words. How does the current study advance those kinds of big debates/discussions in the field? (I've only used mirror / motor theory as an example: I don't necessarily think it's the most suitable one to discuss)

How should we best interpret "acoustic" RDMs, when the morph steps used in the MEG study have been selected via perceptual tasks? The Supplementary Information offers a validation analysis that explains how the morph steps satisfactorily capture within-continuum acoustic differences for the female and the male speakers separately – that is adequate to explain the correspondence between acoustics and step numbers within a continuum, per speaker. But there will be multiple differences between the speakers (as shown in the pitch divergence analysis) and their stimulus continua that are not captured by using the speaker-specific morph steps: point 9 on the male speaker continuum will be acoustically different from point 9 on the female speaker continuum. Moreover, step 9 vs step 16 on the female-speaker continuum will not represent the equivalent acoustic differences as step 9 vs step 16 on the male speaker continuum. It is therefore not surprising that the categorical and acoustic RDMs are so similar based on how they were defined, while an acoustic model based on absolute acoustic distances between the stimuli across both speakers would perhaps capture variance that is more associated with acoustics. It would be interesting to hear more from the authors about why they did not indeed pursue analysis models that included both speakers, in order to capture more variance in perception and acoustics within the RDMs.

(Remarks on code availability)
I inspected what code was available, and listened to some of the experimental stimuli.

Reviewer #2

(Remarks to the Author)
The manuscript reports findings from an MEG study on neural representations of phonemes and prosody. A single monosyllabic word was manipulated to vary along a VOT continuum varying the initial phoneme from /b/ or /p/ in 61 steps. In addition, the pitch contour of the word was manipulated to range from question to statement along 61 steps. For each subject, 25 stimuli were selected (5 phoneme steps, 5 prosody steps) through a staircase procedure. Using time-resolved Representational Similarity Analysis, the authors identified neural regions whose activity matched the similarities among phoneme and prosody levels, both in terms of acoustics and in terms of performance-based categories. They then developed a novel method (multivariate transfer entropy) to quantify the transfer of information across different regions over time. Results reveal the involvement of the ventral stream in the processing of phonemes, and both ventral and dorsal streams for prosody, with transfer of information from posterior to anterior regions. Acoustic information was represented earlier than categories, but the two involved overlapping regions of activity. These results challenge serial hierarchical models of speech perception by revealing both joint and distinct representations of phonemes and prosody, with information being shared bidirectionally to and from primary auditory areas.

Overall, I believe these findings contribute to current models of speech perception, in particular by addressing the neural processing of prosody, which is understudied. The methodology is sound and involved careful acoustic manipulations of the stimuli, which are well described across the paper and the supplementary materials. I particularly appreciated that the authors validated the assumption that both phonemes and prosody were perceived categorically by fitting psychometric curves, as this is well established for phonemes, but less so for prosody. I do not have enough familiarity with RSA and mTE analyses to judge whether they are used appropriately in this study. These analyses were generally well explained in the Results section (though see some clarification questions below), while their description in the Method was very technical and likely only understandable to readers with sufficient mathematical knowledge of these analyses and related terminology.

I only have one primary concern with the overall design. The paper claims to advance current models of speech processing by revealing the neural representations of phoneme and prosody and their abstraction from acoustics to categories. However, given the use of a single phoneme continuum (/b/ to /p/ through VOT) and a single aspect of prosody (question to statement through pitch contour), the generalizability of these findings to broader phonemic and prosodic processing is rather limited. The authors acknowledge the limited generalizability of only focusing on two specific features (VOT and pitch contour) in the discussion. However in my opinion the main issue is not just focusing on these two features, but only using a single exemplar to assess broader phonemic and prosodic representations in the brain.

I think the study's conclusions would be made stronger if the design had included at least two exemplars, such as two phoneme continuums (even if both ranging through VOT, such as /b/-/p/ and /d/-/t/) and two aspects of prosody varying in pitch contour (question-statement, and potentially two different pitch accent types, assuming they would lead to categorical perception). Then, if the neural representations of the two phoneme exemplars and the two prosody exemplars are more similar to each other than they are across phoneme/prosody representations (e.g., if both prosodic exemplars involve both dorsal and ventral streams, while both phoneme exemplars involve only the ventral stream), this would greatly help to generalize to phonemic and prosodic processing more broadly.

While I think the current data is still interesting enough to be published, I believe that this is an important limitation that should be further discussed and that may limit the breadth of the theoretical claims that can be drawn from this data.

Minor comments / clarification questions:

Lines 194-197: It would be helpful to further describe the primary principles behind mTE and transfer entropy for readers unfamiliar with these analyses.

Line 198: please rephrase the sentence starting with "To avoid mixing information over time…". I could not follow what information the authors refer to here, and how they addressed this.

Line 519: please clarify what is meant by "the principles of a type-1 index-1 sequence"

Lines 525-527: please clarify or rephrase "After one-sixth of the stimuli (i.e., twice per stimulus and task in random order)...". Why were responses only probed twice per stimulus? What was randomized, the block order or the stimulus order?

Lines 643-649: what was the random effect structure of the hierarchical linear models?

(Remarks on code availability)

Reviewer #3

(Remarks to the Author)
The study used MEG to explore the brain's processing of prosodic and segmental phonology. It has interesting findings, involving a great spatial overlap for suprasegmental and segmental perception with some differences, lingering acoustic representations in higher-level processing, and reverberating effects from lower to higher levels of processing in the ventral auditory stream.

Major comments

I miss an explicit research question and purpose of the study in the introduction, let alone a hypothesis. Ideally, there should be a theoretically motivated research question and/or hypothesis within the first paragraph(s).

The lack of a theoretically motivated guiding research question carries over to the interpretation and discussion of the results. Due to this, the results presentation and interpretation feels a bit incoherent and scattered.

The part of the last paragraph of the introduction starting with "We show that both phonemic and prosodic information…" is reporting the results. I do not know if this is the style of the journal, but it feels out of place here to me. It looks like a summary of the results before the results have been presented.

Minor comments

It would be good to have a short simple description of the transfer entropy measure since it is non-standard. The authors also present it as novel. It is thoroughly described in a deeper methods section at the end but it would be helpful to have a short statement/presentation at an earlier point, to understand the data analysis.

When you write PAC, I wonder how exact the localization is. Does the activity seem to stem from the auditory core? Heschl's gyrus is a complex structure involving different subareas and often even several gyri with the distribution of primary and secondary processing still being investigated. Perhaps "auditory cortex / AC" or "superior temporal plane" or similar would be a bit safer here.

(Remarks on code availability)

Version 1:

Reviewer comments:

Reviewer #1

(Remarks to the Author)
Thank you to the authors for addressing my comments so clearly in their revision. I find the paper to be much clearer and coherent, and have no further queries.

Just a couple of small points about wording:
- Page 8, line 168: "while dorsal stream areas remained silent". I would recommend re-wording for better accuracy, e.g. "while dorsal areas showed no significant representations of phonemic information"
- Page 9, lines 217-219: "Interactions between posterior temporal areas tended to occur with shorter time lags (within about 50 ms), while their influence on the aSTS (especially, the left aSTS) lasted longer (up to 108 ms)." Perhaps you can clarify the wording such that "their influence" refers to the influence of posterior temporal areas on anterior STS, rather than the influence of *the interactions between* posterior temporal areas* on aSTS (as the wording currently suggests). Please excuse me if this is my error.

(Remarks on code availability)
N/A

Reviewer #2

(Remarks to the Author)
The authors significantly improved the discussion of theoretical background and addressed my concerns about limited generalizability. All of my minor comments and clarification questions were addressed as well. I have no further comments.

(Remarks on code availability)

Reviewer #3

(Remarks to the Author)

The authors have successfully addressed my concerns. The manuscript now reads much better, and the results make more theoretical sense. I only have three comments and a few thoughts on how the authors' results and interpretation can explain some previous findings below.

Three comments:

Auditory cortices --> auditory cortex

656: Mbelt --> MBelt
You could also consider spelling out auditory core, medial belt, lateral belt, and parabelt. I have no strong preference.

Figure 4: Just an observation: In the 3D rendering, the right hemisphere looks like the left hemisphere and vice versa. It is OK, since it says “RH” and “LH,” so I leave it to the authors to decide whether they want to keep the figure as it is (it is probably easier to draw the interconnections on rostrally facing brains, which requires the less intuitive rendering on the “wrong” hemisphere), or if they want to change it.

And a few interesting links to previous research:

399-401: “On top of these subtle differences in the AC and pSTS, prosody categories extended markedly more anteriorly along the ventral stream than phoneme categories, including the aSTS (Fig. 5b,d).”
That is an interesting observation. Previous research has observed that the cortical thickness of the left anterior STS correlated with individuals’ predictive use of prosodic cues. That seems to support the authors’ interpretation. As regards the observation of a network supporting prosodic processing that would involve the right IFG, again, the cortical thickness of the right pars triangularis correlates inversely with pitch discrimination. The thinner cortex is likely an effect of more extensive white-matter connections along the lines the authors describe.

(Remarks on code availability)

Reviewer #4

(Remarks to the Author)
I was asked by the editors to comment on the MEG methods, and I will limit my review to this aspect. There is no indication that the experimental design might not be suited for a MEG experiment, counterbalancing and randomization of the motor mapping ensure unbiased data. MEG data acquisition followed a typical protocol, the parameters and steps used for preprocessing are also common. The filter properties are sufficiently described. I see no issues in the source reconstruction. The RSA procedures, both in the ROI-based and the searchlight-based analysis follow common procedures in the field. The procedures underlying the linear models are clearly described. The mTE method is beyond my expertise, I cannot comment on this.

Major comment

I understand that the authors might want to use NNLS to ensure positive weights on the RDMs, but I do not see why using a max(·,0) operator to zero negative $R2$ that could result from NNLS fitting. It is convenient, but how is it justified? How would not including this step affect the results? Would all major conclusions still hold, and could the step thus be evaded?

Minor comment

The authors should describe the ICA procedure better: how many components were removed per subject on average, and how were these components selected.

(Remarks on code availability)

Open Access This Peer Review File is licensed under a Creative Commons Attribution 4.0 International License, which permits use, sharing, adaptation, distribution and reproduction in any medium or format, as long as you give appropriate credit to the original author(s) and the source, provide a link to the Creative Commons license, and indicate if changes were made.
In cases where reviewers are anonymous, credit should be given to 'Anonymous Referee' and the source.
The images or other third party material in this Peer Review File are included in the article's Creative Commons license, unless indicated otherwise in a credit line to the material. If material is not included in the article's Creative Commons license and your intended use is not permitted by statutory regulation or exceeds the permitted use, you will need to obtain permission directly from the copyright holder.
To view a copy of this license, visit https://creativecommons.org/licenses/by/4.0/

Response to reviews

We are very grateful to the reviewers for their thoughtful and constructive comments on our work. We have carefully addressed each of the points below as well as in the manuscript, which we believe has strongly improved in clarity and overall strength thanks to your input. Two key revisions should be highlighted already here:

[1] Following the suggestion of all reviewers to better theoretically embed and streamline our research goals and findings, we substantially revised the Introduction and Discussion. We now articulate the theoretical backbone of our study—serial hierarchical processing models of speech perception—much more clearly. We explain that our study (i) explicitly probes the spatiotemporal assumptions of these models, and does so (ii) by focusing not only on phonemes (as typically done) but also on prosody, an aspect of speech which has been largely understudied. As a consequence of these clarifications, the multiple ways in which our findings challenge these traditional views gain more impact and visibility, and are now discussed in a much more concise and integrated way.

[2] These revisions may also alleviate the generalisability concern of our study, which arose from investigating a single exemplar of a phonemic and prosodic cue. Given that our primary goal was to probe the predictions of traditional models using two explicitly different types of linguistic elements (in terms of timescale, acoustic cue and function), and given that the results for both elements challenge these predictions in similar ways, our approach already makes a significant step towards generalisation. In fact, adding more exemplars would not change our main conclusion that the existing model needs to be revised. We agree, however, that further steps are needed in future studies that aim to establish general neural processing schemes for phonemes and prosody, which we now acknowledge in the discussion.

Below, we address all of the reviewers' comments one-by-one. The reviewers' original remarks are shown in **black bold** font, and our responses are provided in blue. The original text from the manuscript appears in *black italics*, while new text added in the revision is in *blue italicised* font.

Reviewer #1 (Remarks to the Author):

Baek and colleagues present a combined MEG and behavioural study of speech perception in which they use time-resolved MEG data to interrogate the neural representations of phonemes and prosodic contours, based on voice-onset-time (/b/ vs. /p/) and pitch contour (question vs. statement), respectively. The analyses delineate findings indicating largely overlapping representations of stimulus acoustics and perceived categories for both phonemes and prosody, as well as some relative distinctions between the two aspects of the spoken words (e.g. representation of prosody but not phonemes in inferior frontal and premotor cortices). A multivariate information transfer analysis revealed further differences in the patterning of notionally bottom-up and top-down transfers between regions of interest for phonemic and prosodic cues, respectively.

I find the paper to be very clearly written and all of the methods and findings to be carefully and clearly expressed. The study design and analysis implementation are rigorous and harness methods effectively to offer new insights onto the dual streams posited to support speech perception in the brain. I only have a couple of comments:

Thank you for your positive evaluation of our manuscript and your helpful suggestions regarding the manuscript's structure, and the empirical and theoretical embedding of our results. We followed your suggestions closely, as we will explain in more detail, below.

I felt that having the outline of the findings in the opening sections, followed by the detailed reporting of the results, and another summary in the Discussion, made the text feel somewhat dense and repetitive. If possible, a more integrated reporting of the results would help to make the paper more concise and readable.

Thank you for highlighting this point. We have considerably shortened the summary in the Introduction, focusing on our key findings and main conclusion rather than enumerating the detailed results. In addition, we have substantially re-written large portions of the Discussion to present a more integrative view on the theoretical contributions of our results. We believe these revisions make the manuscript more synthesised and concise, and improve its overall readability.

> *Introduction (lines 84-96):*
> *Here, we show the abstraction and propagation of both phonemes and prosody along the dual speech streams. Notably, our results indicate that the acoustic-to-categorical transformation is not exactly serial as proposed by classical models[6,9–11]. Instead, the two levels of abstraction are processed largely in parallel across time and space. The convergence of these findings for both linguistic elements points to a shared principle of representational abstraction. Marked differences are the spatially broader categorical representations of prosody compared to phonemes along both steams, and the differently weighted contributions of posterior temporal areas. This suggests partially distinct abstraction mechanisms that warrant confirmation in future studies. Together, our findings demonstrate that the brain abstracts both phonemes and prosody through parallel processing of acoustic and categorical information within the dual-stream networks, while flexibly adapting regional involvement and information flow to meet the demands posed by the respective cues and linguistic functions.*
>
> *Discussion (lines 314-331):*
> *Successful speech perception requires transforming continuously variable acoustic signals into perceptually distinct categories. Here, we studied the neural basis of this perceptual transformation for phonemes and prosody, two fundamental building blocks of speech that rely on different acoustic cues and contribute to different aspects of linguistic meanings. Using behavioural psychophysics and MEG, combined with time-resolved RSA and mTE analysis, we found that both phonemic and prosodic representations*

underwent an acoustic-to-categorical abstraction along the ventral stream, with additional involvement of the dorsal stream for prosody. However, contrary to predictions of classical hierarchical processing models, the two abstraction levels significantly overlapped in time and space, indicating their parallel rather than serial processing. The convergence of this finding for both linguistic elements points to a shared principle of representational abstraction. On the other hand, while acoustic representations of phonemes and prosody were similarly distributed across the temporal cortex, their categorical representations markedly differed in spatial extent: Phoneme categories were represented in bilateral posterior temporal areas, with enhanced representations in the left pSTS under top-down focus on phonemes. In contrast, prosody categories were more widespread, including the bilateral aSTS and right PMC. These results suggest partially distinct abstraction mechanisms within the dual streams depending on the type of cue and linguistic function of phonemes and prosody.

(For further changes in the Discussion, we would like to refer the reviewer directly to the manuscript.)

A more emphatic account of the novelty of this study and greater depth of analysis of the theoretical implications would help readers. For example, the finding that representations of phonemic categories do not extend to premotor and inferior frontal cortex – while prosodic categories do - calls to mind the discussions of around 10-15 years ago around the engagement of motor responses for speech perception, where some studies implicated (pre)motor responses in the phonemic analysis of spoken words. How does the current study advance those kinds of big debates/discussions in the field? (I've only used mirror / motor theory as an example: I don't necessarily think it's the most suitable one to discuss)

Thank you for this suggestion. We have made significant revisions throughout our manuscript to better convey the novelty of our findings and their theoretical implications. For example, we have strengthened the Introduction, which now focuses more clearly on the assumptions of traditional serial hierarchical processing models of speech perception (e.g., Hickok & Poeppel, 2007; de Heer et al., 2017; Schirmer & Kotz, 2006) and highlight that these models have come under scrutiny in recent years. As we now explain, the novelty of our study is exactly to (i) probe this traditional view, and to do so by (ii) expanding the investigation of categorical perception beyond phonemes, to prosody that has been understudied. We believe that these clarifications not only increase the theoretical depth of our manuscript but also provide readers with better guidance to grasp the multiple facets of our findings.

Introduction (lines 44-70):
[…] Classical hierarchical processing models propose that, within this dual-stream architecture, continuously variable acoustic signals are serially transformed into more abstract linguistic representations across both time and space[6,9–11]*. That is, speech is first acoustically analysed in sensory areas and then represented as discrete categories of linguistic elements in higher-order*

regions, including lateral and anterior temporal[12–14], motor[15,16] or inferior frontal regions[17,18].

However, this simple serial hierarchy has recently been challenged in several ways—for example, by studies showing categorical representations in the primary auditory cortex[19–21], or direct encoding of abstract linguistic information in the superior temporal cortex, bypassing sensory areas[22]. Although evidence from functional neuroimaging or electrophysiology has provided some indications of where and when acoustic-phonetic[23–26] or categorical[17,21,27–29] features of speech are represented in the brain, studies have often been unable to effectively dissociate these two representations, leaving it elusive how the acoustic-to-categorical mapping actually occurs. […]

Notably, almost all studies seeking to uncover the acoustic-to-categorical mapping in speech have focused on phonemes[15,17,20,29,33], leaving one important aspect of speech largely unexplored: speech prosody. Compared to phonemes, prosody is not only less well established in its categorical perception but is also characterised by distinct acoustic cues; whether it engages similar neural processing is therefore still an open question. […]

Also in the Discussion, we now articulate the novelties and theoretical contributions of our study much more clearly: Our study (i) demonstrates the neural encoding of not only phonemic but also prosodic categories, (ii) is one of the few that truly captures categorical representation beyond acoustics and links it to behaviour (which previous studies have rarely done), and (iii) revises the traditional model by revealing parallel, rather than serial, acoustic and categorical processing. In particular, we now provide a more thorough discussion of which results align or conflict with theoretical predictions—including those of the motor theory of speech perception (Liberman & Mattingly, 1985), and propose potential explanations for the conflicts based on the literature.

Discussion (lines 332-433):

(To avoid a lengthy reply here, please refer to the revised manuscript for this section.)

de Heer, W. A., Huth, A. G., Griffiths, T. L., Gallant, J. L., & Theunissen, F. E. (2017). The hierarchical cortical organization of human speech processing. *Journal of Neuroscience*, *37*(27), 6539-6557.

Hickok, G., & Poeppel, D. (2007). The cortical organization of speech processing. *Nature Reviews Neuroscience*, *8*(5), 393-402.

Liberman, A. M., & Mattingly, I. G. (1985). The motor theory of speech perception revised. *Cognition*, *21*(1), 1-36.

Schirmer, A., & Kotz, S. A. (2006). Beyond the right hemisphere: brain mechanisms mediating vocal emotional processing. *Trends in Cognitive Sciences*, *10*(1), 24-30.

How should we best interpret "acoustic" RDMs, when the morph steps used in the MEG study have been selected via perceptual tasks? The Supplementary Information offers a validation analysis that explains how the morph steps

satisfactorily capture within-continuum acoustic differences for the female and the male speakers separately – that is adequate to explain the correspondence between acoustics and step numbers within a continuum, per speaker. But there will be multiple differences between the speakers (as shown in the pitch divergence analysis) and their stimulus continua that are not captured by using the speaker-specific morph steps: point 9 on the male speaker continuum will be acoustically different from point 9 on the female speaker continuum. Moreover, step 9 vs step 16 on the female-speaker continuum will not represent the equivalent acoustic differences as step 9 vs step 16 on the male speaker continuum. It is therefore not surprising that the categorical and acoustic RDMs are so similar based on how they were defined, while an acoustic model based on absolute acoustic distances between the stimuli across both speakers would perhaps capture variance that is more associated with acoustics. It would be interesting to hear more from the authors about why they did not indeed pursue analysis models that included both speakers, in order to capture more variance in perception and acoustics within the RDMs.

Thank you for this very thoughtful question. It is true that the *absolute* acoustic values of the per-participant selected stimuli may have differed between the two speakers: For example, point 9 on the male and female continua may not have been physically identical in terms of VOT or pitch slope. However, it must be noted that the *difference* between step 9 and step 16, that is, their physical/acoustic similarity which is relevant in the RSA, *is* (close to) identical across speakers. This is because, for example, in the phoneme continuum, VOT increased linearly by 1ms per morph step (please see *Stimuli* in Methods).

What is much more important than the acoustic similarity *between* speakers, is that *within* each speaker, the acoustic difference between all consecutive morph steps in a continuum (i.e., p1-p2, p2-p3, p3-p4, and p4-p5) is *identical*, while the perceptual difference between these steps, is *not* identical. In other words, what we model and compare is a linear acoustic change vs. a non-linear perceptual change—the very nature of categorical perception. The similarity between the categorical and acoustic RDMs is primarily driven by the fact that higher acoustic values on a continuum naturally increase the likelihood to be perceived as a different category. Including both speakers in one RDM would not solve that issue. On the contrary, because this RDM would also include acoustic differences between speakers, the subtle acoustic-phonetic dissimilarities within a speaker would be overpowered by dissimilarities between speakers (in speaking rate and voice), and would no longer allow us to clearly track the acoustic-to-categorical transformation. Instead, such an RDM would lead us to find speaker-sensitive areas.

Perhaps the term “acoustic RDM” is misleading, as we are technically looking into “acoustic-phonetic” features which are somewhat different between speakers and contexts (co-articulation) (e.g., Fitch & Giedd, 1999) and which listeners quickly adapt to (sometimes called “speaker-normalization”; e.g., Johnson & Sjerps, 2021; Liu & Liu, 2025; Sjerps et al., 2019; Tang et al., 2017). We deliberately centred stimuli on participants’ categorical boundaries per speaker, to account for these speaker differences, and test the feature-range that gives rise to the acoustic-to-categorical transition in phonemes and prosody, irrespective of the absolute acoustic

values. We have clarified our rationale behind the stimulus creation and our modelling of acoustic RDM in the Methods:

> *Methods (lines 519-528):*
> *Out of the 61 x 61 steps, 5 x 5 morph levels were selected for each participant based on two behavioural pre-tests […] to identify five levels of VOT and pitch contour centred on individual category boundaries. The distance between the five levels was selected to ensure that the difficulty of categorising phonemes and prosody was matched. […] This approach ensured that stimuli covered the feature-range that gave rise to the acoustic-to-categorical transition in phonemes and prosody in each speaker and participant, irrespective of the absolute acoustic values.*

Fitch, W. T., & Giedd, J. (1999). Morphology and development of the human vocal tract: A study using magnetic resonance imaging. *The Journal of the Acoustical Society of America*, *106*(3), 1511-1522.

Johnson, K., & Sjerps, M. J. (2021). Speaker normalization in speech perception. *The Handbook of Speech Perception*, 145-176.

Lammert, A. C., & Narayanan, S. S. (2015). On short-time estimation of vocal tract length from formant frequencies. *PloS one*, *10*(7), e0132193.

Liu, S., & Liu, X. (2025). Context influence on speech perception: evidence for acoustic-level mechanism across the voice onset time continuum. *NeuroImage*, *310*, 121140.

Tang, C., Hamilton, L. S., & Chang, E. F. (2017). Intonational speech prosody encoding in the human auditory cortex. *Science*, *357*(6353), 797-801.

Reviewer #2 (Remarks to the Author):

The manuscript reports findings from an MEG study on neural representations of phonemes and prosody. A single monosyllabic word was manipulated to vary along a VOT continuum varying the initial phoneme from /b/ or /p/ in 61 steps. In addition, the pitch contour of the word was manipulated to range from question to statement along 61 steps. For each subject, 25 stimuli were selected (5 phoneme steps, 5 prosody steps) through a staircase procedure. Using time-resolved Representational Similarity Analysis, the authors identified neural regions whose activity matched the similarities among phoneme and prosody levels, both in terms of acoustics and in terms of performance-based categories. They then developed a novel method (multivariate transfer entropy) to quantify the transfer of information across different regions over time. Results reveal the involvement of the ventral stream in the processing of phonemes, and both ventral and dorsal streams for prosody, with transfer of information from posterior to anterior regions. Acoustic information was represented earlier than categories, but the two involved overlapping regions of activity. These results challenge serial hierarchical models of speech perception by revealing both joint and distinct representations of phonemes and prosody, with information being shared bidirectionally to and from primary auditory areas.

Overall, I believe these findings contribute to current models of speech

perception, in particular by addressing the neural processing of prosody, which is understudied. The methodology is sound and involved careful acoustic manipulations of the stimuli, which are well described across the paper and the supplementary materials. I particularly appreciated that the authors validated the assumption that both phonemes and prosody were perceived categorically by fitting psychometric curves, as this is well established for phonemes, but less so for prosody. I do not have enough familiarity with RSA and mTE analyses to judge whether they are used appropriately in this study. These analyses were generally well explained in the Results section (though see some clarification questions below), while their description in the Method was very technical and likely only understandable to readers with sufficient mathematical knowledge of these analyses and related terminology.

Thank you so much for your positive feedback and for offering very helpful advice on how to improve our manuscript. We agree that some explanations in the Methods section are rather technical, but we believe that this precision is necessary for future replication. To still improve accessibility to a broad readership, we tried to clarify the Results section even further (see below). We address each of your points in detail below.

I only have one primary concern with the overall design. The paper claims to advance current models of speech processing by revealing the neural representations of phoneme and prosody and their abstraction from acoustics to categories. However, given the use of a single phoneme continuum (/b/ to /p/ through VOT) and a single aspect of prosody (question to statement through pitch contour), the generalizability of these findings to broader phonemic and prosodic processing is rather limited. The authors acknowledge the limited generalizability of only focusing on two specific features (VOT and pitch contour) in the discussion. However in my opinion the main issue is not just focusing on these two features, but only using a single exemplar to assess broader phonemic and prosodic representations in the brain.

I think the study's conclusions would be made stronger if the design had included at least two exemplars, such as two phoneme continuums (even if both ranging through VOT, such as /b/-/p/ and /d/-/t/) and two aspects of prosody varying in pitch contour (question-statement, and potentially two different pitch accent types, assuming they would lead to categorical perception). Then, if the neural representations of the two phoneme exemplars and the two prosody exemplars are more similar to each other than they are across phoneme/prosody representations (e.g., if both prosodic exemplars involve both dorsal and ventral streams, while both phoneme exemplars involve only the ventral stream), this would greatly help to generalize to phonemic and prosodic processing more broadly.

While I think the current data is still interesting enough to be published, I believe that this is an important limitation that should be further discussed and that may limit the breadth of the theoretical claims that can be drawn from this data.

Thank you for your valid concern, which we are taking very seriously. We agree that stronger conclusions may have been possible with more exemplars. However, we would like to clarify that the primary aim of this study was *not* to identify and compare the general neural processing schemes for phonemes and prosody. Rather, our goals were (i) to determine whether continuously variable acoustic signals are mapped onto discrete categories in ways assumed by a classical model (i.e., serial hierarchical processing), and (ii) to probe this model by investigating the neural categorical perception of not only phonemes but also prosody—another fundamental building block of speech that operates at a longer timescale and has been rarely explored. One novelty of our study is that the dynamic acoustic-to-categorical mapping along the ventral and dorsal speech stream is not exactly serial, but parallel in time and space, for both linguistic elements. We believe that this convergence across phonemes and prosody, even if stemming from single exemplars, is a valid step towards generalisation of our findings. In fact, adding more exemplars would not change our main conclusion that the existing model needs to be rethought.

We realize that the original text left room to understand the phoneme-vs-prosody comparison as our primary (and only) goal, while probing theoretical predictions with two explicitly different types of linguistic elements was not expressed clearly enough. We have therefore made significant revisions throughout our manuscript to better communicate the priorities of our research goals. This includes (1) the revision of the Title to mark the acoustics-to-category transfer the primary aim and outcome; (2) the more explicit presentation of the classical theory, its predictions, and our research questions in the Introduction (see also the Abstract); and (3) the more streamlined Discussion of our results in direct relation to these predictions, including a revised statement on the generalisability of our findings.

> *Title (lines 1-2):*
> *Old: Shared and distinct representational dynamics of phonemes and prosody in ventral and dorsal speech streams*
> *New: Dynamic acoustic-to-categorical representations of phonemes and prosody along ventral and dorsal speech streams*
>
> *Introduction (lines 44-70):*
> *[…] Classical hierarchical processing models propose that, within this dual-stream architecture, continuously variable acoustic signals are serially transformed into more abstract linguistic representations across both time and space[6,9–11]. That is, speech is first acoustically analysed in sensory areas and then represented as discrete categories of linguistic elements in higher-order regions, including lateral and anterior temporal[12–14], motor[15,16] or inferior frontal regions[17,18].*
> *However, this simple serial hierarchy has recently been challenged in several ways […] Although evidence from functional neuroimaging or electrophysiology has provided some indications of where and when acoustic-phonetic[23–26] or categorical[17,21,27–29] features of speech are represented in the brain, studies have often been unable to effectively dissociate these two representations, leaving it elusive how the acoustic-to-categorical mapping actually occurs. […]*

Notably, almost all studies seeking to uncover the acoustic-to-categorical mapping in speech have focused on phonemes[15,17,20,29,33], leaving one important aspect of speech largely unexplored: speech prosody. Compared to phonemes, prosody is not only less well established in its categorical perception but is also characterised by distinct acoustic cues; whether it engages similar neural processing is therefore still an open question. […]

Discussion (lines 332-386):

(To avoid a lengthy reply here, please refer to the revised manuscript for these sections)

Discussion (lines 449-458):
Before closing, several caveats regarding our findings should be noted. First, we examined only a single example of a phonemic and a prosodic cue—/b/-/p/ along the VOT continuum, and statement-question pitch contours—limiting the generalisabilty of our findings to other distinctive features. However, these exemplars, that deliberately tap into different timescales to probe and extend prevailing knowledge, already revealed aspects of speech processing that cannot be accounted for by existing models, such as categorical representations in early sensory areas and parallel acoustic and categorical processing in both time and space. Future studies on other phonemic and prosodic cues will help to substantiate the observed representational dynamics and further estimate the extent to which traditional serial hierarchical processing models needs to be revised. […]

Minor comments / clarification questions:

Lines 194-197: It would be helpful to further describe the primary principles behind mTE and transfer entropy for readers unfamiliar with these analyses.

Thank you for highlighting this point. We have added a description of the primary principles behind transfer entropy and mTE in the Results.

Results (lines 192-196):
Next, we employed mTE analysis to elucidate how phonemic and prosodic information flows between the cortical areas identified above[36,37]. mTE is a multivariate extension of transfer entropy that allows the assessment of directed representational transfer between regions based on neural RDMs. This approach is similar to previous methods using Granger causality[40,41] but offers advantages in capturing non-linear interactions.

Line 198: please rephrase the sentence starting with "To avoid mixing information over time…". I could not follow what information the authors refer to here, and how they addressed this.

Thank you for your suggestion. We agree that the original phrasing was unclear. The phrase was intended to contrast how neural RDMs were computed in the "time-

resolved RSA" and the "mTE analysis". In the time-resolved RSA, neural RDMs were computed by averaging distance estimates within 24-ms time windows. That is, a neural RDM at a given time point already contained "information" (or "representation") from future time points (e.g., a neural RDM at 12 ms resulted from averaging across sample points spanning 0 to 24 ms). Therefore, using the same set of neural RDMs in the mTE analysis was problematic, as leakage of information from the future could spuriously increase the strength of directed information transfer, especially at smaller time lags. We thus re-computed neural RDMs based on individual time samples in the mTE analysis "to avoid mixing information over time". We have rephrased the original sentence accordingly in the revised manuscript.

> *Results (lines 198-202):*
> *[…] In these ROIs, we first re-generated time series of neural RDMs—this time without applying sliding windows—to ensure that the RDM at each time point was not influenced by preceding or subsequent time points. Specifically, we re-computed dissimilarities between MEG response patterns evoked by the five phoneme or prosody levels at each time sample from 0 to 800 ms after stimulus onset. […]*

Line 519: please clarify what is meant by "the principles of a type-1 index-1 sequence"

Thank you for bringing this to our attention. A "type-1 index-1 sequence" is a method of ordering experimental stimuli to cancel out carry-over effects of the preceding stimulus on the processing (e.g., categorical perception) of the present stimulus (Nonyane & Theobald, 2007). In principle, each unique stimulus (e.g., phoneme level 1) in this sequence is allowed to follow every other unique stimulus (i.e., phoneme levels 2, 3, 4, and 5), including itself, for the same number of times. However, we write "principles of" because it is mathematically impossible to create a perfect type-1 index-1 sequence based on 5 phoneme or prosody levels. To still capitalize on the benefits of a balanced stimulus order, we allowed one or two breaches of the rule per block in order to form a "type-1 index-1-like" sequence. Sequences varied across speakers and runs to further minimise the influence of these subtle breaches. We have clarified this in the Methods section.

> *Methods (lines 545-549):*
> *[…] After the practice trials,* 25 *unique* stimuli *(i.e., 5 x 5 morph levels of the speaker) were presented pseudorandomly following the principles of a type-1 index-1 sequence to prevent carry-over and position effects[63]. Specifically, the stimuli were ordered such that, ideally, each morph level was followed by every other morph level, including itself, twice per block. Stimulus order varied across speakers and runs.*

Nonyane, B. A. S., & Theobald, C. M. (2007). Design sequences for sensory studies: Achieving balance for carry-over and position effects. *British Journal of Mathematical and Statistical Psychology*, *60*(2), 339-349.

Lines 525-527: please clarify or rephrase "After one-sixth of the stimuli (i.e.,

twice per stimulus and task in random order)...". Why were responses only probed twice per stimulus? What was randomized, the block order or the stimulus order?

Thank you for these questions. We collected behavioural responses only in one-sixth of the trials because response trials took considerably longer (due to prompt presentation and pause after the button press) than regular trials in which participants mentally categorised the stimuli without giving an overt response. To maximise the overall number of trials (and SNR) in the experiment, we considered 1/6th (i.e., 10 trials for each of the 5 phoneme and prosody levels per speaker) sufficient for fitting reliable psychometric functions (please see Fig. 1c,d). With "in random order" we mean that response trials occurred randomly within each of the blocks, to ensure that listeners remain attentive throughout the experiment. We have rephased the section accordingly.

> *Methods (lines 554-565):*
> *[…] Participants were asked to report their percept by pressing a button on a response box corresponding to the on-screen category labels, but only in one-sixth of the trials (i.e., 10 responses for each of the five phoneme and prosody levels per speaker). This was to maximise the overall number of trials (and signal-to-noise ratio) within the recording session, by saving time spent on responses and pause intervals, and while acquiring sufficient behavioural data for fitting reliable psychometric functions. Response trials occurred randomly within each block to ensure participants remained attentive throughout the experiment. Additionally, response buttons were randomly assigned on each response trial to avoid confounding category labels with preparatory motor activity. The labels "Bar" and "Paar" (or "statement" and "question") were presented on either the left or right side of the screen after stimulus offset, with their positions balanced across stimuli, tasks, and speakers.*

Lines 643-649: what was the random effect structure of the hierarchical linear models?

We used the term "hierarchical linear modelling" not to refer to linear mixed-effects models, but to describe our approach of comparing the variance explained by full and null or reduced linear models. No random effects were included in these linear models. We recognise that this term could cause confusion, and have thus replaced it with "nested model comparisons" in the revised manuscript.

> *Methods (lines 681-682, 699-701):*
> *We quantified neural representations of phonemes and prosody by nested model comparisons. Specifically, three types of nested linear models were built […]*
> *The estimated R^2 from the nested models was used to assess general representations, as well as unique acoustic and categorical representations (Fig. 2f), as shown below: […]*

Reviewer #3 (Remarks to the Author):

The study used MEG to explore the brain's processing of prosodic and segmental phonology. It has interesting findings, involving a great spatial overlap for suprasegmental and segmental perception with some differences, lingering acoustic representations in higher-level processing, and reverberating effects from lower to higher levels of processing in the ventral auditory stream.

We appreciate your helpful feedback on the organisation and flow of our manuscript. We have carefully considered your comments and addressed each of your points individually below. We believe that the manuscript has greatly improved in clarity and coherence thanks to your input.

Major comments

I miss an explicit research question and purpose of the study in the introduction, let alone a hypothesis. Ideally, there should be a theoretically motivated research question and/or hypothesis within the first paragraph(s).

Thank you for this thoughtful feedback. We have significantly revised the Introduction which now spells out the theoretical framing, the guiding research questions and predictions of our study much more explicitly. We essentially ask (i) exactly where and when in the established ventral and dorsal speech streams, continuously variable acoustic signals are mapped onto discrete linguistic categories, and (ii) whether this acoustic-to-categorical mapping is similar between two explicitly different types of categories—phonemes (relying on transient cues) and prosody (relying on temporally more extended cues). Hence, the novelties of our study are that we directly probe the predictions of a classical model on how information passes through the streams—serial hierarchical processing theory—and we expand the investigation of categorical perception beyond phonemes, to prosody.

> *Introduction (lines 43-70):*
> *Speech processing is known to involve distributed frontotemporal areas, organised into ventral and dorsal streams[6–8]. Classical hierarchical processing models propose that, within this dual-stream architecture, continuously variable acoustic signals are serially transformed into more abstract linguistic representations across both time and space[6,9–11]. That is, speech is first acoustically analysed in sensory areas and then represented as discrete categories of linguistic elements in higher-order regions, including lateral and anterior temporal[12–14], motor[15,16] or inferior frontal regions[17,18].*
> *However, this simple serial hierarchy has recently been challenged in several ways […] Although evidence from functional neuroimaging or electrophysiology has provided some indications of where and when acoustic-phonetic[23–26] or categorical[17,21,27–29] features of speech are represented in the brain, studies have often been unable to effectively dissociate these two representations, leaving it elusive how the acoustic-to-categorical mapping actually occurs. […]*
> *Notably, almost all studies seeking to uncover the acoustic-to-categorical mapping in speech have focused on phonemes[15,17,20,29,33], leaving*

one important aspect of speech largely unexplored: speech prosody. Compared to phonemes, prosody is not only less well established in its categorical perception but is also characterised by distinct acoustic cues; whether it engages similar neural processing is therefore still an open question. […]

The lack of a theoretically motivated guiding research question carries over to the interpretation and discussion of the results. Due to this, the results presentation and interpretation feel a bit incoherent and scattered.

We fully agree with this observation, thank you. The revision of the Introduction (see above) has had direct repercussions on the Discussion, which we have substantially revised. More precisely, we now discuss our results more directly in relation to predictions of serial hierarchical models, leading us to conclude that our data challenge this account in multiple ways, consistently for phonemes and prosody. In the second half of the Discussion, we now link the differences between phonemes and prosody more explicitly to previous reports of dissociated functional pathways to pSTS and AC (Hamilton et al., 2021; Gnataneja et al., 2025), and preferred temporal integration windows along the ventral (Norman-Haignere et al., 2022) and dorsal stream (Assaneo & Poeppel, 2018). We believe that these revisions now present our data in a much more integrated and coherent way.

Discussion (lines 314-444):

(To avoid a lengthy reply here, please refer to the revised manuscript for this section of the Discussion)

Assaneo, M. F., & Poeppel, D. (2018). The coupling between auditory and motor cortices is rate-restricted: Evidence for an intrinsic speech-motor rhythm. *Science advances*, *4*(2), eaao3842.

Gnanateja, G. N., Rupp, K., Llanos, F., Hect, J., German, J. S., Teichert, T., ... & Chandrasekaran, B. (2025). Cortical processing of discrete prosodic patterns in continuous speech. *Nature Communications*, *16*(1), 1947.

Hamilton, L. S., Oganian, Y., Hall, J., & Chang, E. F. (2021). Parallel and distributed encoding of speech across human auditory cortex. *Cell*, *184*(18), 4626-4639.

Norman-Haignere, S. V., Long, L. K., Devinsky, O., Doyle, W., Irobunda, I., Merricks, E. M., ... & Mesgarani, N. (2022). Multiscale temporal integration organizes hierarchical computation in human auditory cortex. *Nature Human Behaviour*, *6*(3), 455-469.

The part of the last paragraph of the introduction starting with "We show that both phonemic and prosodic information…" is reporting the results. I do not know if this is the style of the journal, but it feels out of place here to me. It looks like a summary of the results before the results have been presented.

Indeed, it is journal style to summarise the key findings at the end of the Introduction. However, we agree that our original summary was too dense and lengthy. We now

limit ourselves to anticipating the main conclusions of the study, which we then return to in more detail in the Discussion section.

> *Introduction (lines 84-96):*
> *Here, we show the abstraction and propagation of both phonemes and prosody along the dual speech streams. Notably, our results indicate that the acoustic-to-categorical transformation is not exactly serial as proposed by classical models[6,9–11]. Instead, the two levels of abstraction are processed largely in parallel across time and space. The convergence of these findings for both linguistic elements points to a shared principle of representational abstraction. Marked differences are the spatially broader categorical representations of prosody compared to phonemes along both steams, and the differently weighted contributions of posterior temporal areas. This suggests partially distinct abstraction mechanisms that warrant confirmation in future studies. Together, our findings demonstrate that the brain abstracts both phonemes and prosody through parallel processing of acoustic and categorical information within the dual-stream networks, while flexibly adapting regional involvement and information flow to meet the demands posed by the respective cues and linguistic functions.*

Minor comments

It would be good to have a short simple description of the transfer entropy measure since it is non-standard. The authors also present it as novel. It is thoroughly described in a deeper methods section at the end but it would be helpful to have a short statement/presentation at an earlier point, to understand the data analysis.

Thank you for your suggestion. We now provide a brief description of the mTE measures in the Results.

> *Results (lines 192-196):*
> *Next, we employed mTE analysis to elucidate how phonemic and prosodic information flows between the cortical areas identified above[36,37]. mTE is a multivariate extension of transfer entropy that allows the assessment of directed representational transfer between regions based on neural RDMs. This approach is similar to previous methods using Granger causality[40,41] but offers advantages in capturing non-linear interactions.*

When you write PAC, I wonder how exact the localization is. Does the activity seem to stem from the auditory core? Heschl's gyrus is a complex structure involving different subareas and often even several gyri with the distribution of primary and secondary processing still being investigated. Perhaps "auditory cortex / AC" or "superior temporal plane" or similar would be a bit safer here.

Indeed, in this study, the PAC was defined by aggregating "A1" and the surrounding belt areas ("Mbelt", medial belt; "Lbelt", lateral belt; "PBelt", parabelt) based on the

HCP-MMP parcellation (Glasser et al., 2016). Given the functional complexity of these areas, we agree that referring to this aggregation as PAC could be misleading. Following your suggestion, we have replaced “primary auditory cortex / PAC” with “auditory cortex / AC” throughout the manuscript. In addition, we now spell out the list of HCP-MMP parcels used to define each ROI in the Methods.

> *Methods (lines 652-657):*
> *Neural RDMs were primarily constructed from source activity in 10 predefined ROIs (Fig. 2a). The ROIs were defined based on a multimodal cortical parcellation[69] to be comparable in size and spatially distinct to minimise cross-talk. They covered areas previously shown to be involved in the processing of phonemes and prosody[7,8,28], including the bilateral IFG (44, 45, IFSa, IFSp), PMC (6r, 6v, PEF, 55b), AC (A1, Mbelt, LBelt, PBelt), pSTS (STSdp, STSvp) and aSTS (STSda, STSva). […]*

Glasser, M. F., Coalson, T. S., Robinson, E. C., Hacker, C. D., Harwell, J., Yacoub, E., ... & Van Essen, D. C. (2016). A multi-modal parcellation of human cerebral cortex. *Nature*, *536*(7615), 171-178.

Response to reviews

We thank the three reviewers for their time and efforts reviewing our revised work, and the fourth reviewer for assessing the soundness of our MEG data analyses. We have carefully addressed all of the reviewers' comments point-by-point below as well as in the manuscript, including the concern raised by the fourth reviewer regarding zeroing negative R^2 values in the RSA.

The reviewers' original remarks are shown in **black bold** font, and our responses are provided in blue. The original text from the manuscript appears in *black italics*, while new text added in the revision is in *blue italicised* font.

Reviewer #1 (Remarks to the Author):

Thank you to the authors for addressing my comments so clearly in their revision. I find the paper to be much clearer and coherent, and have no further queries.

Thank you for your positive feedback and for your constructive suggestions throughout the review process, which have significantly improved the clarity and coherence of our manuscript.

Just a couple of small points about wording:
- Page 8, line 168: "while dorsal stream areas remained silent". I would recommend re-wording for better accuracy, e.g. "while dorsal areas showed no significant representations of phonemic information"

Thank you for your suggestion. We have rephrased the sentence as follows:

> *Results (lines 167-169):*
> *Fig. 3a shows that phonemic information was represented exclusively in ventral stream ROIs in both hemispheres, while dorsal areas showed no significant representations of phonemic information. [...]*

- Page 9, lines 217-219: "Interactions between posterior temporal areas tended to occur with shorter time lags (within about 50 ms), while their influence on the aSTS (especially, the left aSTS) lasted longer (up to 108 ms)." Perhaps you can clarify the wording such that "their influence" refers to the influence of posterior temporal areas on anterior STS, rather than the influence of *the interactions between* posterior temporal areas* on aSTS (as the wording currently suggests). Please excuse me if this is my error.

Thank you for bringing this to our attention. We have clarified "their influence" in the revised manuscript:

> *Results (lines 218-220):*

[...] Interactions between posterior temporal areas tended to occur with shorter time lags (within about 50 ms), while the influence of posterior temporal areas on the aSTS (especially, the left aSTS) lasted longer (up to 108 ms). [...]

Reviewer #2 (Remarks to the Author):

The authors significantly improved the discussion of theoretical background and addressed my concerns about limited generalizability. All of my minor comments and clarification questions were addressed as well. I have no further comments.

Thank you for your careful reading of our revision and for your thoughtful comments throughout the process. We are pleased that the expanded theoretical discussion and the clarifications regarding generalizability have addressed your concerns.

Reviewer #3 (Remarks to the Author):

The authors have successfully addressed my concerns. The manuscript now reads much better, and the results make more theoretical sense. I only have three comments and a few thoughts on how the authors' results and interpretation can explain some previous findings below.

We appreciate your positive assessment of our revision and your insightful thoughts throughout the review process, which helped to elevate the theoretical embedding and implications of our work. We have addressed each of your comments below.

Three comments:

Auditory cortices --> auditory cortex

Thank you for suggestion. We have carefully reviewed the entire manuscript and replaced “auditory cortices” with “auditory cortex”. We have also ensured that the other brain regions are referred to in the singular in the same sentence for consistency.

Results (lines 140-144):
As illustrated in Fig. 2a, neural dissimilarities were quantified in 10 predefined regions of interest (ROIs) across the two hemispheres, including the inferior frontal gyrus (IFG) and premotor cortex (PMC) in the dorsal stream, as well as the auditory cortex (AC) and the posterior (pSTS) and anterior superior temporal sulcus (aSTS) in the ventral stream [...]

656: Mbelt --> MBelt
You could also consider spelling out auditory core, medial belt, lateral belt, and parabelt. I have no strong preference.

Thank you for spotting the typo. It has been corrected in the revised manuscript.

> *Methods (lines 661-664):*
> *[...] They covered areas previously shown to be involved in the processing of phonemes and prosody[7,8,28], including the bilateral IFG (44, 45, IFSa, IFSp), PMC (6r, 6v, PEF, 55b), AC (A1, MBelt, LBelt, PBelt), pSTS (STSdp, STSvp) and aSTS (STSda, STSva). [...]*

Regarding the names of AC subregions, we have retained "MBelt, LBelt, and PBelt" as these are the labels defined in the HCP-MMP1.0 atlas used in our study. For readers wishing to replicate our analysis, we believe it is clearer to maintain the original labels from the atlas.

Figure 4: Just an observation: In the 3D rendering, the right hemisphere looks like the left hemisphere and vice versa. It is OK, since it says "RH" and "LH," so I leave it to the authors to decide whether they want to keep the figure as it is (it is probably easier to draw the interconnections on rostrally facing brains, which requires the less intuitive rendering on the "wrong" hemisphere), or if they want to change it.

Thank you for your comment on Figure 4. For visualization purposes, both hemispheres were mirrored (reflected across the midline) during figure preparation in Adobe Illustrator.

In an initial version, the two hemispheres were plotted facing in opposite directions, which made it difficult to arrange the arrows in a clean and organized manner (see Figure R1).

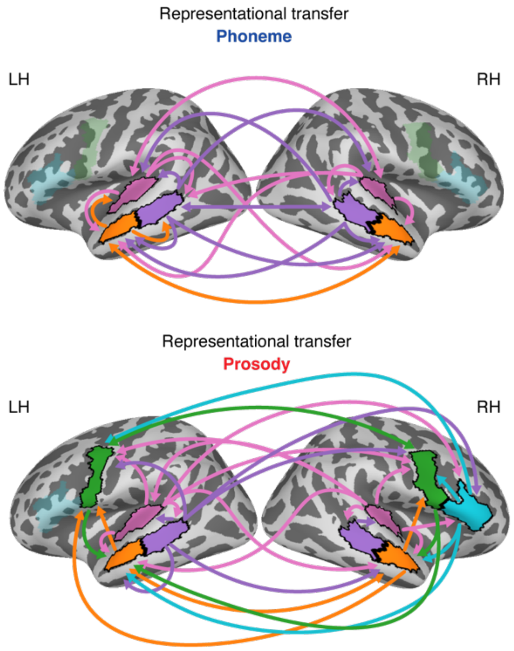

Figure R1. Preliminary schematic illustrations of phonemic and prosodic representational transfer (corresponding to **Fig. 4a,d** in the manuscript).

To minimize potential confusion, we have re-arranged Figure 4a and d, so that the left hemisphere is shown on the right side and the right hemisphere on the left (without flipping individual hemispheres). We believe this layout improves readability and effectively illustrates the connections between regions. We have also clarified this in the figure legend:

> *Figure 4:*
> ***a*,*d*, *[...] Arrows indicate directed representational transfer from the source to the target ROIs, colour-coded according to the source ROIs. For visualization, the left hemisphere is shown on the right side and the right hemisphere on the left.***
> ***b*,*c*,*e*,*f*, *[...]***

And a few interesting links to previous research:

399-401: "On top of these subtle differences in the AC and pSTS, prosody categories extended markedly more anteriorly along the ventral stream than phoneme categories, including the aSTS (Fig. 5b,d)."
That is an interesting observation. Previous research has observed that the cortical thickness of the left anterior STS correlated with individuals' predictive use of prosodic cues. That seems to support the authors' interpretation. As regards the observation of a network supporting prosodic processing that would involve the right IFG, again, the cortical thickness of the right pars triangularis correlates inversely with pitch discrimination. The thinner cortex is likely an effect of more extensive white-matter connections along the lines the authors describe.

Thank you for pointing us to these fascinating connections to the structural literature. We are aware of this important work from Lund. Unfortunately, the strict word limits do not allow us to go into more detail in the manuscript, but we included Novén et al. (2021, Brain Research) in the Discussion.

> *Discussion (lines 407-410):*
> *[...] Overall, both properties align with characteristic differences between phonemes—which are brief and often lack concrete meanings[2,32]—and prosody—which generally spans longer durations and imparts morphosyntactic[54] or pragmatic meanings such as the speaker's intention[3]. [...]*

Reviewer #4 (Remarks to the Author):

I was asked by the editors to comment on the MEG methods, and I will limit my review to this aspect. There is no indication that the experimental design might not be suited for a MEG experiment, counterbalancing and randomization of the motor mapping ensure unbiased data. MEG data acquisition followed a typical protocol, the parameters and steps used for preprocessing are also common. The filter properties are sufficiently described. I see no issues in the source reconstruction. The RSA procedures, both in the ROI-based and the searchlight-based analysis

follow common procedures in the field. The procedures underlying the linear models are clearly described. The mTE method is beyond my expertise, I cannot comment on this.

We appreciate the time spent assessing our MEG data acquisition and analysis protocols. We are pleased that our experimental design, preprocessing pipeline, source reconstruction, and RSA procedures were found to be sound and clearly described. We have addressed your remaining points regarding the RSA and ICA one-by-one below.

Major comment

I understand that the authors might want to use NNLS to ensure positive weights on the RDMs, but I do not see why using a max($\cdot$,0) operator to zero negative R2 that could result from NNLS fitting. It is convenient, but how is it justified? How would not including this step affect the results? Would all major conclusions still hold, and could the step thus be evaded?

Thank you for your thoughtful question regarding the handling of negative R^2 values. Our decision to clip negative R^2 values to zero was a deliberate choice motivated by both (1) the interpretation of model fits in RSA and (2) the requirements of our statistical inference procedure.

In our RSA pipeline, we used unbiased estimators (Crossnobis distance) combined with non-negative constraints (NNLS). In this context, a negative R^2 indicates that the model explains less variance that a null model (i.e., mean). In practical terms, such values do not reflect meaningful "negative explainability", but rather the absence of explanatory power (e.g., Kriegeskorte et al., 2008, *Frontiers in system neuroscience*; Yamins et al., 2014, *PNAS*). Therefore, values below zero are functionally equivalent to zero in terms of interpretability.

From a statistical perspective, this step is required for the Fisher-Z transformation applied prior to group-level inference. Specifically, we converted R^2 values to a correlation-like measure ($r = \sqrt{R^2}$), which is basically not defined for negative values. The Fisher-Z transformation was then used to improve normality, stabilise variance, and make the values more comparable across participants and time points. Skipping this step would violate key assumptions of the subsequent t-tests (used in cluster-based inference), thereby reducing statistical sensitivity.

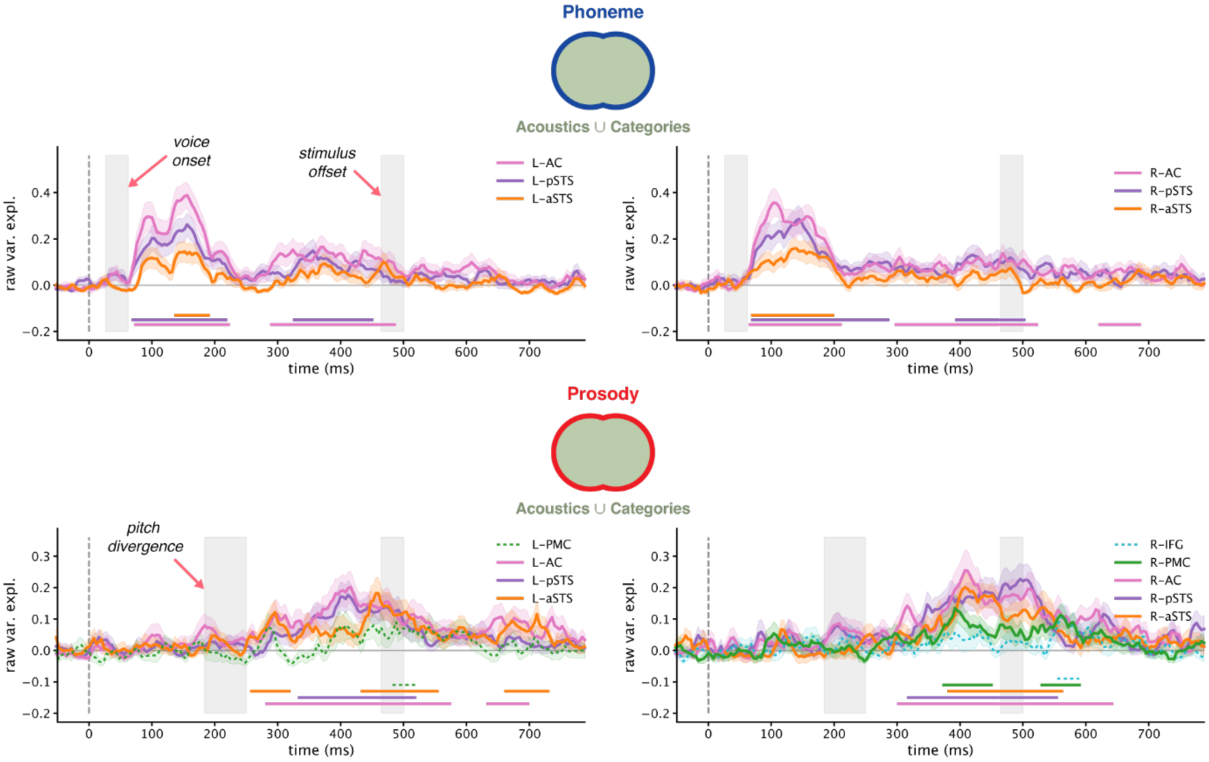

Figure R2. Phonemic and prosodic representations from the re-analysis. This re-analysis followed the same protocol used to generate **Fig. 3** in the manuscript, but without zeroing negative R-squared values and without Fisher-Z transformation. Solid lines denote significant clusters, while dashed lines indicate clusters that no longer reached significance in this re-analysis.

In Figure R2, we still provide the results of a re-analysis (corresponding to **Fig. 3** in the manuscript) without clamping negative R^2 values and without applying the Fisher-Z transformation (i.e., $\hat{R}_G^2 := \hat{R}_{\text{full}}^2 - \hat{R}_{\text{null}}^2$; cf. Equation 3). As shown, the overall results remain consistent with the original findings, suggesting that our main conclusions do not depend on this step. However, prosodic representations in the left PMC and right IFG no longer reach significance (both are marginally significant; L-PMC: $p = 0.054$; R-IFG: $p = 0.070$). This change does not seem to be driven by differences in the underlying response profiles, which remain nearly identical, but may rather stem from the increased variance and skewedness in the untransformed R^2 values.

In fact, the distribution of the untransformed R^2 values in Figure 2 shows a heavy, long tail, as illustrated in Figure R3 (left column). Please note that negative values are effectively zero, with magnitudes ranging between 10^-15 and 10^-16, indicating that our $\max(\cdot, 0)$ operator in Equation 3 did not actually bias the R^2 values. However, some R^2 values exceeded 1, which suggests the possibility that R^2 might be overestimated due to the subtraction of negative $\hat{R}_{\text{null}}^2$ values. When we transformed these R^2 values via the Fisher Z transformation (after clipping negative values), the distributions became less skewed (Figure R3, right column; excluding $\hat{R}_G^2$ values larger than 1).

Taken together, we believe that zeroing negative R^2 values and applying the Fisher-Z transformation do not introduce bias or alter the direction of effects, but instead

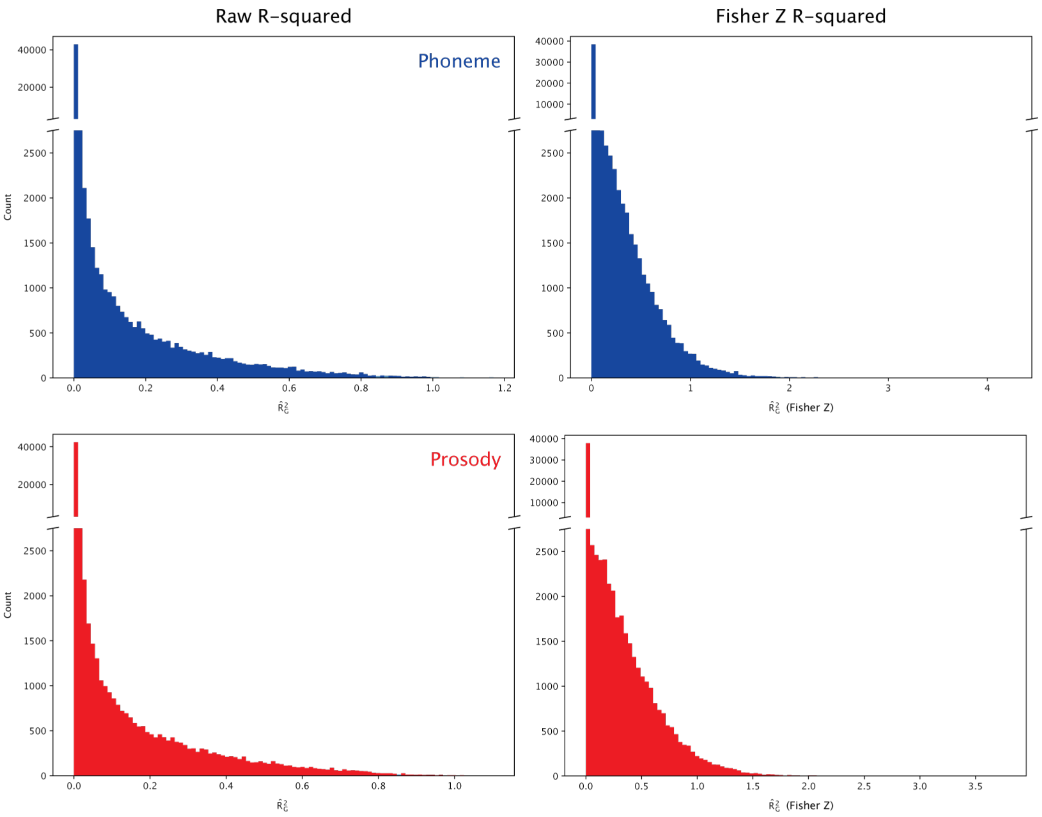

Figure R3. Distributions of raw (left column) and Fisher-Z transformed (right column) R-squared values from phoneme (top row) and prosody (bottom row) analyses. The R-squared values correspond to Figure R2, collapsed across participants, ROIs, and time points.

provide a statistically well-founded way to stabilise variance and maintain sensitivity for detecting representational effects at the group level.

In the revised manuscript, we have provided a rationale for zeroing negative R^2 values in terms of RSA interpretability:

> *Methods (lines 712-716):*
> *[...], NNLS can yield a value below zero when the predictors explain less variance in the dependent variable than its mean, due to the non-negative weight constraint. Such values do not reflect meaningful negative explainability, but rather the absence of explanatory power, functionally equivalent to zero in terms of RSA interpretability. In our case, [...]*

Minor comment

The authors should describe the ICA procedure better: how many components were removed per subject on average, and how were these components selected.

Thank you for highlighting this point. We have updated the Methods section to include the specific criteria for component selection and the distribution of rejected components across participants.

Methods (lines 614-622):

The filtered signals were then downsampled to 250 Hz and subjected to independent component analysis (ICA) to remove physiological artefacts. The ICA decomposition was computed separately for each participant using data highpass-filtered at 1 Hz and concatenated across all six runs to improve solution quality; these 1-Hz filtered signals were used exclusively for this step and were not included in further analyses. Artefactual components (e.g., eye movements and heartbeats) were semi-automatically identified using the find_bads_eog and find_bads_ecg functions, combined with visual inspection, and subsequently rejected (median = 4 components, 10th-90th percentiles: 3-6).

[...]
